# Alternative Splicing Regulated by Butyrate in Bovine Epithelial Cells

**DOI:** 10.1371/journal.pone.0039182

**Published:** 2012-06-14

**Authors:** Sitao Wu, Congjun Li, Wen Huang, Weizhong Li, Robert W. Li

**Affiliations:** 1 Center for Research in Biological Systems, University of California San Diego, San Diego, California, United States of America; 2 USDA-ARS, Bovine Functional Genomics Laboratory, Beltsville, Maryland, United States of America; 3 Department of Genetics, North Carolina State University, Raleigh, North Carolina, United States of America; Georgia Institute of Technology, United States of America

## Abstract

As a signaling molecule and an inhibitor of histone deacetylases (HDACs), butyrate exerts its impact on a broad range of biological processes, such as apoptosis and cell proliferation, in addition to its critical role in energy metabolism in ruminants. This study examined the effect of butyrate on alternative splicing in bovine epithelial cells using RNA-seq technology. Junction reads account for 11.28 and 12.32% of total mapped reads between the butyrate-treated (BT) and control (CT) groups. 201,326 potential splicing junctions detected were supported by ≥3 junction reads. Approximately 94% of these junctions conformed to the consensus sequence (GT/AG) while ∼3% were GC/AG junctions. No AT/AC junctions were observed. A total of 2,834 exon skipping events, supported by a minimum of 3 junction reads, were detected. At least 7 genes, their mRNA expression significantly affected by butyrate, also had exon skipping events differentially regulated by butyrate. Furthermore, COL5A3, which was induced 310-fold by butyrate (FDR <0.001) at the gene level, had a significantly higher number of junction reads mapped to Exon#8 (Donor) and Exon#11 (Acceptor) in BT. This event had the potential to result in the formation of a COL5A3 mRNA isoform with 2 of the 69 exons missing. In addition, 216 differentially expressed transcript isoforms regulated by butyrate were detected. For example, Isoform 1 of ORC1 was strongly repressed by butyrate while Isoform 2 remained unchanged. Butyrate physically binds to and inhibits all zinc-dependent HDACs except HDAC6 and HDAC10. Our results provided evidence that butyrate also regulated deacetylase activities of classical HDACs via its transcriptional control. Moreover, thirteen gene fusion events differentially affected by butyrate were identified. Our results provided a snapshot into complex transcriptome dynamics regulated by butyrate, which will facilitate our understanding of the biological effects of butyrate and other HDAC inhibitors.

## Introduction

Alternative splicing (AS) is an important driving force behind vast protein diversity and the evolution of phenotypic complexity in mammals. AS generates various protein isoforms from single genes with different biological properties [1] and plays a critical role in development and disease [2]–[4]. Furthermore, AS is under strict regulatory control, including an accurate recognition of the splice junction that defines intron and exon boundary [5]. AS is mediated by elaborate molecular machinery, the spliceosome, which consists of at least 5 small nuclear RNAs (snRNAs) and over 100 accessory proteins [6]. Mutations in the core element of the spliceosome cause disease [7]. Up to 94% of human genes are estimated to undergo AS [4]; hence, aberrant AS is frequently associated with numerous human diseases [1]. Therefore, splicing modulation has been touted as a therapeutic means for treating genetic diseases caused by splicing mutations [8].

Butyrate is a preferred substrate for gut epithelial cells. In ruminants, butyrate contributes to 70% of energy metabolism. In monogastric species, butyrate also plays an important role in energy metabolism in the hindgut [9]. Moreover, butyrate acts as a histone deacetylase inhibitor and can induce apoptosis and inhibit cell proliferation *in vitro* [10]. As a signaling molecule, butyrate induces a profound change in gene expression and results in a significant change in transcript abundance of ∼50% genes transcribed in the transcriptome of epithelial cells [11]. The effect of butyrate on growth rate of ovarian carcinoma cells *in vitro* is associated with a significant decrease of proto-oncogene MYC. Down-regulation of MYC is accomplished at least partially by accelerated MYC mRNA degradation and by inhibiting splicing [12]. Furthermore, butyrate is known to have an impact on AS of vascular endothelial growth factor (VEGF) gene [13]. Butyrate significantly increases the expression levels of both mRNA and protein of transcript variants that are anti-angiogenic in human lung endothelial cells while the pro-angiogenic isoform is not detectable [13]. However, the effect of butyrate on AS has not been systematically investigated in cattle. In this study, we presented evidence that butyrate can influence AS using high-throughput transcriptome technology and bioinformatics.

## Results

### 1. Splice Junction Inference

The total number of reads mapped to the bovine genome (UMD3.1) using the Genomic Short-read Nucleotide Alignment Program (GSNAP) [14] were 52.25 and 47.73 million for the butyrate-treated (BT) and control (CT) groups, respectively (Table 1). For the sake of simplicity, only reads uniquely mapped to the genome (i.e., hitting only one locus) were counted. Among them, junction reads–those spanning exon-exon junctions with at least 8 bp of overhangs in each exon–accounted for 11.28 and 12.32% for the butyrate and control groups, respectively (*P*<0.001). These junction coverage results were in agreement with a published report [15], which shows 10–16% reads containing splice junctions in breast tumor samples. A total of 799,589 potential splicing junction sites were detected. Of them, 201,326 splice junction sites were supported by a minimum of 3 junction reads (mean; *N*=8). Approximately 94% of all splicing sites conformed to the consensus sequence, GT/AG while ∼3% of junction sites belonged to the most common non-consensus splice junction GC/AG. The percentage of GC/AG junction observed in bovine epithelial cells was higher than that observed in humans and other species [16] where the GT-GC conversation represents only a small fraction at the donor sites (<1%). No AT/AC splice junction sites were observed in the bovine epithelial cell in this study (Table 1).

**Table 1:** Summary of junction reads.

	Butyrate	Control	*P* value
Total mapped reads	52,250,742±8,350,021	47,631,511±5,010,005	NA
Junction reads %	11.28±0.27	12.32±0.19	0.0007
GT/AG Junction %	93.53±0.83	93.85±0.21	0.4868
GC/AG Junction %	3.08±0.27	2.76±0.12	0.0709
AT/AC Junction %	0.00±0.00	0.00±0.00	NA
Other Junctions %	3.39±0.57	3.39±0.12	0.9975

The mean length of input reads was 49 bp and two mismatches were allowed by GSNAP. Reads hitting more than one locus in the genome were excluded. Each exon-exon junction was supported by at least one junction (spliced) read with a minimum of eight bases aligned to each exon. Read counts were normalized. Numbers denote mean ± sd (*N*=4).

### 2. Exon Skipping

A total of 2,834 potentially consistent exon skipping events were detected using GSNAP. A minimum of three junction reads (mean across all eight samples tested) supported each of these events. These junction reads were mapped to the same chromosome and the same strand in the correct order within a close proximity of 20,000 bp as defined (consistent), as compared to other common events, such as scrambles, where the two halves of junction reads were mapped to the same chromosome and on the same strand but in the wrong order. The 2,504 events were involved in 2,113 genes. Table 2 lists select exon skipping events with the difference in mean junction read counts greater than 20.0 between butyrate-treated and control groups. The gene expression level of all genes involved in these exon skipping events was significantly regulated by butyrate treatment at a stringent false discovery rate (FDR <0.0001) [11]. For example, collagen, type V, alpha 3 (COL5A3) was significantly up-regulated (∼310 fold) by butyrate (*P*<0.0001 and FDR <0.0001). The mean number of junction reads mapped to exon #8 (donor) and exon#11 (acceptor) in butyrate-treated cells was 16.00 (±5.10, sd) while no junction reads spanning this region (0.00±0.00) were detected in control cells. This event had the potential to result in the formation of an mRNA isoform with 2 of the COL5A3 69 exons missing (exon#9 and exon#10). Quantitative RT-PCR confirmed this event (data not shown). Similarly, Exon#2 of S100A1 gene may be skipped, supported by multiple junction reads in BT, and the resultant new isoform was also differentially regulated by butyrate (Table 2).

**Table 2:** Exon skipping events supported by multiple junction reads detected using GSNAP.

GENE_ID	Gene	Junction	Donor	Acceptor	BT	CT	P value	Exons skipped
ENSBTAG00000020548	ADC	S0136341	–2∶121444188	–2∶121441559	10.25±4.50	0.50±0.58	0.0051	Exon#3
ENSBTAG00000001927	ATP6V1C2	S0315271	–11∶86867132	–11∶86863699	6.00±4.55	0.25±0.50	0.0456	Exon#11
ENSBTAG00000010179	COL5A3	S0120443	–7∶15803726	–7∶15802198	16.00±5.10	0.00±0.00	0.0008	Exon#9 & Exon#10
ENSBTAG00000011998	DUT	S0460161	–10∶62295493	–10∶62292296	0.25±0.50	11.00±4.24	0.0024	Exon#2
ENSBTAG00000034680	FAM131C	S0142521	+2∶136612861	+2∶136616956	7.25±1.89	0.25±0.50	0.0004	Exon#4 & Exoin#5
ENSBTAG00000018169	ITGB4	S0053350	–19∶56477748	–19∶56475490	11.00±3.56	0.00±0.00	0.0008	Exon#32 & Exon#33
ENSBTAG00000005163	S100A1	S0197907	–3∶16816122	–3∶16812906	6.50±1.29	0.00±0.00	0.0001	Exon#2

The number under Butyrate and Control denotes mean counts of junction reads (± sd; *N* =4). Of exon skipping events detected, only those with the difference in the mean read count between the 2 groups greater than 20.0 were listed. All genes in this table were significantly regulated by butyrate at the gene level at a strict false discovery rate (FDR) <0.001.

### 3. Gene Fusion Events Significantly Affected by Butyrate

Only consistent splice junctions–both halves of junction reads mapped to the same chromosome strand in the correct order within a default distance, 20,000 bp–were considered in this study. Translocation (two halves mapped to different chromosomes), inversion (two halves mapped to the same chromosome but on opposite strands), scramble (on the same chromosome strand but in an incorrect order) as well as the genomic deletion [17] were excluded. The local gene fusion events or read-through in the bovine epithelial cells were readily detected by GSNAP. Of these, 13 events were significantly affected by butyrate (*P*<0.05; Table 3). The genes involved in these events were within the genomic interval set by the default parameter, -w =20kb. As the table shows, about half of the events are involved in the members of gene families. For example, the gene fusion event involving keratin 8 (donor gene) and keratin 4 (acceptor gene) was supported by 236 and 165 junction reads for BT and CT groups, respectively (*P*<0.0001). This event was verified independently using RT-PCR. Similarly, a gene fusion between homeobox A6 (HOXA6) and homeobox A5 (HOXA5) was supported by 3.47 and 7.19 junction reads for BT and CT, respectively (*P*<0.05). Interestingly, this gene fusion also started from an alternative 5′ splice junction (donor site), changing the 3′ boundary of the upstream exon.

**Table 3:** Gene fusion events significantly affected by butyrate in the bovine epithelial cells.

Donor Gene	Acceptor Gene	Exon involved (Donor)	Exon involved (Acceptor)	Butyrate (BT)	Control (CT)	*P* value
GNAS3	GNAS	B Exon3 (5)	E Exon6 (13)	311.39±13.62	421.34±17.64	0.0001
KRT8	KRT4	E Exon5 (12)	B Exon 3 (9)	236.01±13.21	165.25±9.62	0.0001
MYL6B	MYL6	B Exon 3 (7)	E Exon 4 (7)	96.29±12.20	134.74±11.28	0.0036
EEF2	ZFR2	E Exon 13 (15)	I Exon16-Exon17 (19)	23.37±6.59	13.69±3.52	0.0411
RBM12	CPNE1	B Exon 1 (3)	E Exon 2 (16)	20.07±4.39	12.08±4.74	0.0483
RNF207	ICMT	B Exon 16 (18)	E Exon7 (7)	13.19±4.10	7.95±0.98	0.0474
HBZ	HBA	E Exon2 (3)	B Exon3 (3)	11.37±1.38	3.86±1.88	0.0007
UBA3	TMF1	E Exon12 (18)	I Exon8-Exon9 (17)	7.42±1.42	3.81±1.86	0.0217
SNORA3	RL27A	M Exon 1 (1)	E Exon4 (5)	5.59±2.26	0.43±0.50	0.0043
HHIPL2	AIDA	I Exon3-Exon4 (9)	I Exon6-Exon7 (10)	4.51±2.38	1.43±0.98	0.0500
HOXA6	HOXA5	I Exon2-Exon3 (3)	B Exon2 (2)	3.47±1.98	7.19±2.20	0.0454
PPP6R2	NCAPH2	I Exon21-Exon22 (22)	B Exon10 (21)	2.63±0.66	12.78±5.90	0.0142
ZNF865	ZNF524	I Exon2-Exon3 (3)	M Exon1 (2)	1.98±0.98	10.28±2.18	0.0004

The number denotes normalized counts of junction reads that support the events (mean ± sd; *N*=4).

**Table 4:** Differentially expressed isoforms detected using MISO in the bovine epithelial cells.

Transcript_ID	Exon	Gene	ΔΨ	Bayes factor	Butyrate	Control	*P* value
ENSBTAT00000055849	Exon1-10	CNST	0.23	1.01E+01	127.5±16.3	288.0±53.4	0.0012
ENSBTAT00000001328	Exon1-8	TCF7	0.24	1.24E+02	409.8±117.5	740.4±84.2	0.0038
ENSBTAT00000002887	Exon1-13	TRPC1	–0.53	2.91E+02	106.1±69.6	287.6±73.2	0.0115
ENSBTAT00000003925	Exon1-10	SESN1	0.30	2.44E+01	124.4±47.5	43.6±21.4	0.0211
ENSBTAT00000004789	Exon1-23	LPHN1	–0.57	1.98E+01	2077.4±203.2	215.3±77.1	0.0000
ENSBTAT00000004832	Exon1-22	XPO4	–0.21	1.95E+05	379.2±34.2	1234.3±117.6	0.0000
ENSBTAT00000043910	Exon1-19	CDC45	–0.41	3.70E+15	308.1±28.4	485.6±71.2	0.0036
ENSBTAT00000005623	Exon1-20	CDC45	–0.59	3.70E+15	186.0±69.2	1677.8±61.3	0.0000
ENSBTAT00000005904	Exon1-16	RGS3	0.20	1.31E+11	298.1±104.9	88.7±56.1	0.0125
ENSBTAT00000061169	Exon1-22	RGS3	0.80	1.31E+11	1348.5±201.0	3809.6±321.8	0.0000
ENSBTAT00000009025	Exon1-11	LGALS9	–0.56	3.53E+26	162.8±20.7	28.3±6.1	0.0000
ENSBTAT00000009005	Exon1-10	LGALS9	–0.34	3.53E+26	263.3±41.7	6.7±6.5	0.0000
ENSBTAT00000010525	Exon1-15	NCF2	0.24	1.29E+20	109.8±18.7	605.2±54.4	0.0000
ENSBTAT00000063771	Exon1-10	NCF2	0.76	1.29E+20	170.9±18.7	447.6±63.5	0.0002
ENSBTAT00000010689	Exon1-17	CLSTN3	0.44	1.12E+01	259.5±138.2	16.5±10.5	0.0127
ENSBTAT00000010687	Exon1-18	CLSTN3	0.56	1.12E+01	5215.3±529.7	22.0±10.6	0.0000
ENSBTAT00000037763	Exon1-4	C1orf144	–0.23	1.87E+02	436.5±99.5	937.6±80.6	0.0002
ENSBTAT00000013080	Exon1-10	NFYA	–0.22	1.91E+01	1163.7±138.5	562.4±138.1	0.0008
ENSBTAT00000014028	Exon1-9	OCIAD1	0.35	2.54E+12	1036.8±83.2	310.9±107.1	0.0000
ENSBTAT00000046355	Exon1-9	OCIAD1	0.65	2.54E+12	1183.9±107.7	2833.6±232.6	0.0000
ENSBTAT00000014097	Exon1-12	NTRK2	0.64	2.59E+11	236.0±13.4	8.8±4.2	0.0000
ENSBTAT00000014288	Exon1-16	TACC2	–0.56	1.00E+12	732.6±123.3	2646.9±179.7	0.0000
ENSBTAT00000061158	Exon1-22	TACC2	–0.34	1.00E+12	3954.1±324.8	928.6±77.7	0.0000
ENSBTAT00000052258	Exon1-14	NUMB	–0.32	3.97E+29	396.6±122.7	1202.9±178.0	0.0003
ENSBTAT00000015508	Exon1-11	NUMB	–0.68	3.97E+29	2654.4±190.3	1423.0±202.5	0.0001
ENSBTAT00000015772	Exon1-9	CCDC24	–0.25	1.20E+01	284.8±36.1	22.2±12.4	0.0000
ENSBTAT00000065091	Exon1-10	NDEL1	–0.33	3.60E+01	1039.2±213.9	510.9±61.5	0.0032
ENSBTAT00000016883	Exon1-9	ZDHHC16	–0.31	4.20E+02	258.2±85.8	448.7±59.0	0.0106
ENSBTAT00000017205	Exon1-20	PRUNE	–0.55	2.81E+11	37.0±4.5	3.7±1.5	0.0000
ENSBTAT00000036664	Exon1-11	PRUNE	–0.45	2.81E+11	76.4±18.7	2.4±1.5	0.0002
ENSBTAT00000018490	Exon1-20	WDR52	0.43	1.39E+01	43.6±6.9	4.9±4.3	0.0001
ENSBTAT00000036545	Exon1-8	GUK1	0.15	8.20E+101	142.9±46.8	771.1±69.6	0.0000
ENSBTAT00000057267	Exon1-14	NASP	–0.20	3.50E+70	730.0±25.2	2444.7±179.7	0.0000
ENSBTAT00000020401	Exon1-16	NASP	–0.80	3.50E+70	766.3±62.3	5612.5±209.0	0.0000
ENSBTAT00000047968	Exon1-8	ZFPM1	–0.31	2.62E+02	595.1±17.9	175.3±41.1	0.0000
ENSBTAT00000066331	Exon1-7	EME2	–0.26	2.58E+01	19.1±14.5	37.9±12.7	0.0996
ENSBTAT00000056488	Exon1-3	NR2F2	–0.32	1.47E+01	24.8±11.0	311.0±64.1	0.0001
ENSBTAT00000023969	Exon1-3	NR2F2	–0.68	1.47E+01	194.4±30.0	534.5±110.8	0.0010
ENSBTAT00000024380	Exon1-7	PAOX	0.22	5.00E+05	644.9±73.7	965.0±103.5	0.0024
ENSBTAT00000026432	Exon1-12	SHC1	0.20	7.80E+106	2466.4±195.7	1403.0±208.4	0.0003
ENSBTAT00000040264	Exon1-13	SHC1	0.80	7.80E+106	4534.0±17.6	7195.6±384.6	0.0001
ENSBTAT00000027531	Exon1-11	LNX1	–0.58	1.25E+01	222.7±17.6	24.0±25.5	0.0000
ENSBTAT00000027603	Exon1-6	BACH2	0.45	1.96E+01	73.6±17.1	13.5±3.3	0.0005
ENSBTAT00000043742	Exon1-13	MX1	0.31	7.46E+01	632.7±160.7	159.4±37.1	0.0012
ENSBTAT00000012035	Exon1-13	MX1	0.69	7.46E+01	5267.2±970.5	298.5±31.4	0.0001
ENSBTAT00000055077	Exon1-8	ACY1	0.22	1.00E+12	174.1±17.6	1041.0±86.8	0.0000
ENSBTAT00000045814	Exon1-14	ACY1	–0.16	1.04E+21	1820.6±200.5	1443.5±122.4	0.0184
ENSBTAT00000056825	Exon1-24	VAV1	0.70	5.67E+03	187.8±36.9	11.8±5.8	0.0001
ENSBTAT00000045636	Exon1-11	RANBP6	–0.20	1.00E+12	2395.3±249.6	1939.1±273.4	0.0488
ENSBTAT00000061564	Exon1-26	RANBP6	–0.80	1.00E+12	7688.9±461.7	17216.6±977.8	0.0000

The number under Butyrate and Control denotes mean counts of junction reads (± sd; *N* =4). Exon denotes the exons in the transcript. Exon 1–17 indicates that this transcript contains exon 1 to exon 17. ΔΨ denotes the difference in mean posterior distribution between two samples. Bayes factor represents the odds of differential expression over no differential expression (mean of all significant comparisons). P values were calculated from junction reads between butyrate-treated and untreated control groups using a modified *t*-test.

### 4. Differentially Expressed Isoforms Induced by Butyrate

**Figure 1:**
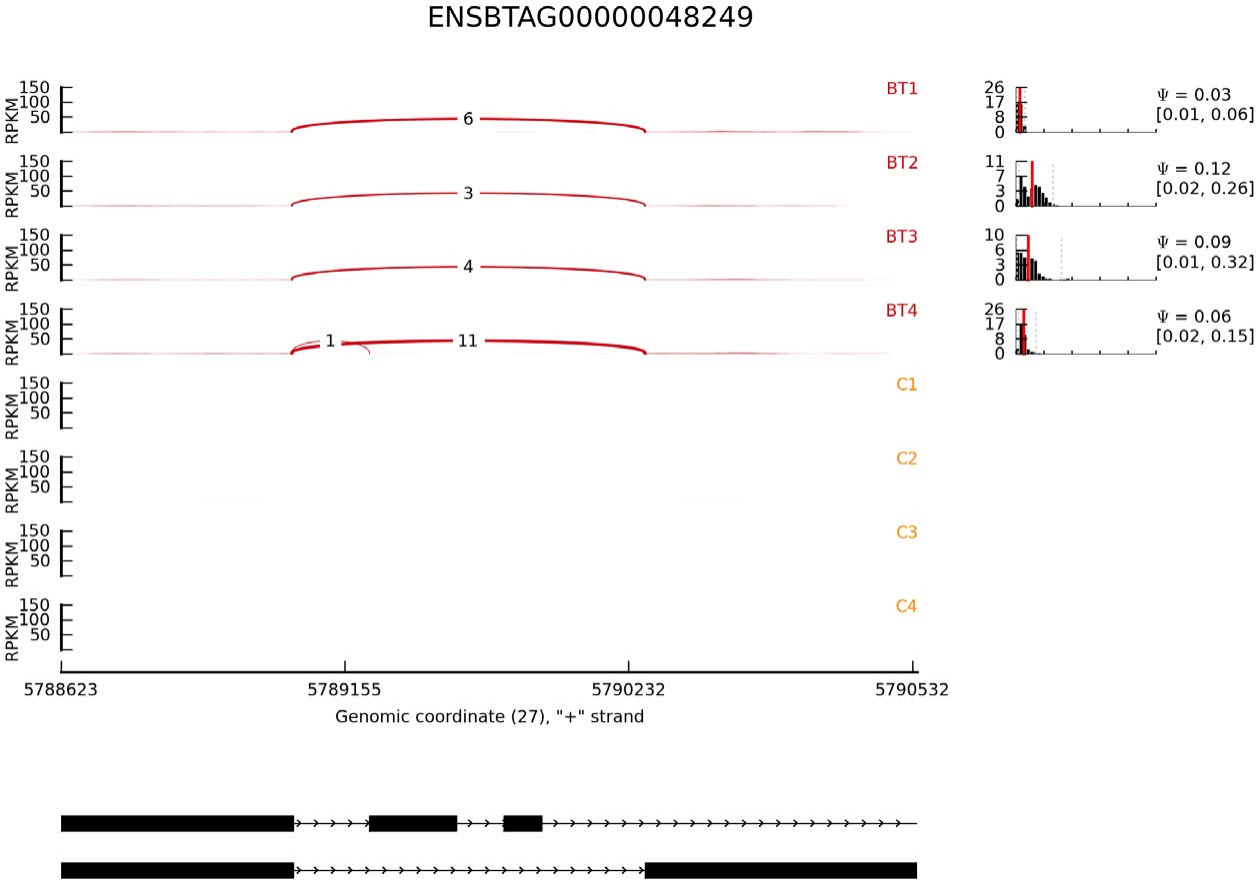
Relative abundance of transcript isoforms of β-defensin 1 gene induced by butyrate in bovine epithelial cells. Reads per kilobase of exon model per million mapped reads (RPKM) were displayed in the Y-axis for each sample tested. BT: butyrate-treated cells. C: untreated control cells. Arcs with numbers represent junction reads. Diagrams below show the gene structures of two transcript isoforms. Left: the posterior distribution with Ψ value.

**Figure 2:**
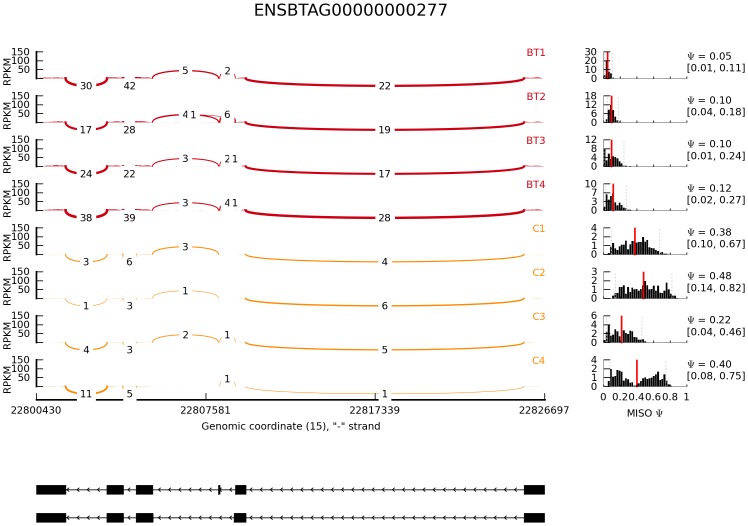
Relative abundance of transcript isoforms of IL-18 gene induced by butyrate in bovine epithelial cells. Reads per kilobase of exon model per million mapped reads (RPKM) were displayed in the Y-axis for each sample tested. BT: butyrate-treated cells. C: untreated control cells. Arcs with numbers represent junction reads. Bold arcs show the junction supported by >10 junction reads. Diagrams below show the gene structures of two transcript isoforms. Left: the posterior distribution with Ψ value.

**Figure 3:**
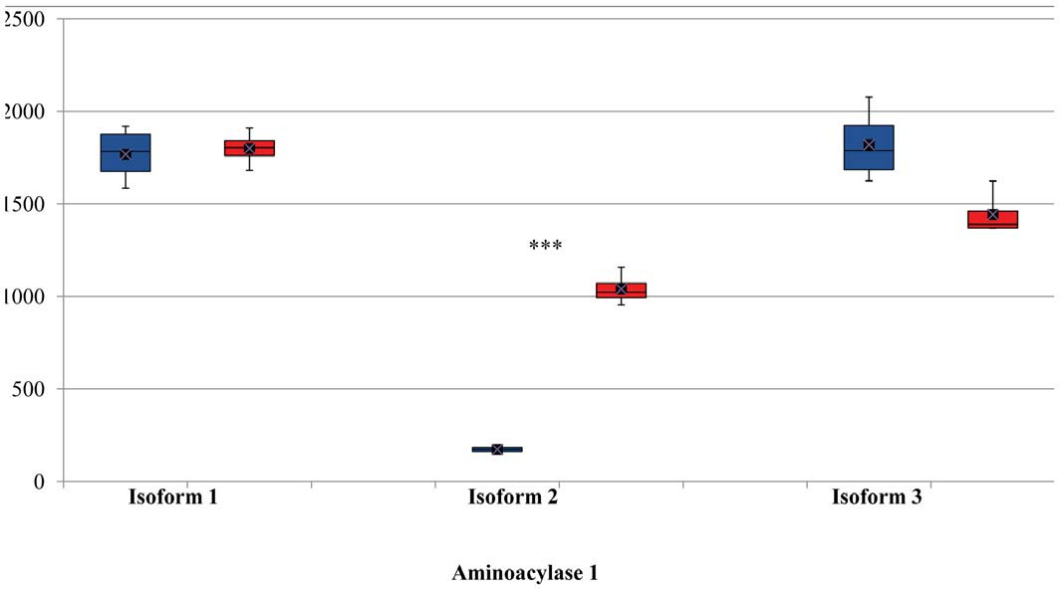
Relative abundance of transcript isoforms of aminoacylase 1 gene. Boxes denote the inter-quartile range between the 1^st^ and 3^rd^ quartiles (25 and 75%, respectively). Blue: butyrate-treated cells; Red: untreated control cells. Y-axis: normalized read counts. ***indicates P<0.0001.

**Figure 4:**
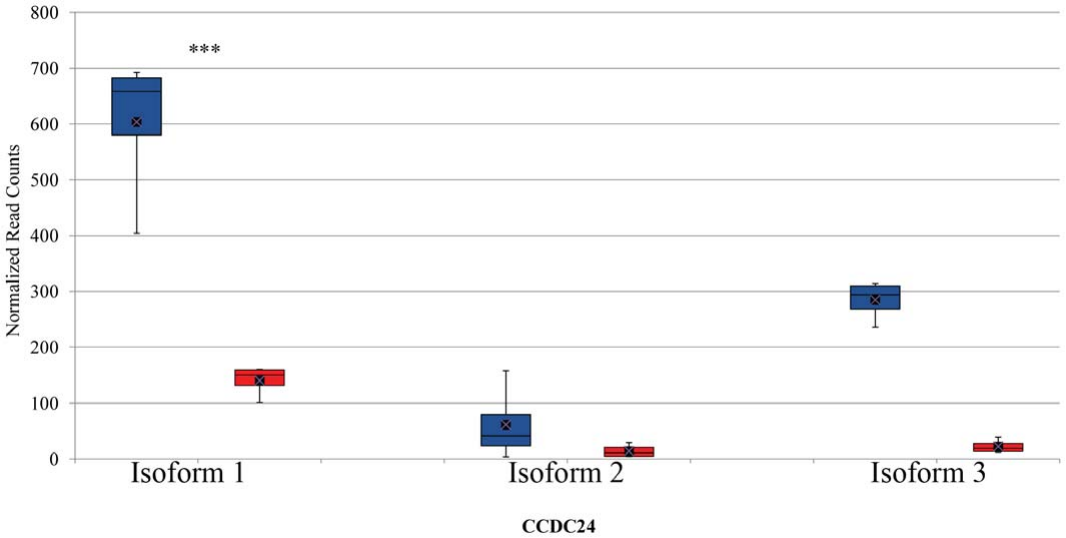
Relative abundance of transcript isoforms of coiled-coil domain containing 24 (CCDC24) gene in bovine epithelial cells. Boxes denote the inter-quartile range between the 1^st^ and 3^rd^ quartiles (25 and 75%, respectively). Blue: butyrate-treated cells; Red: untreated control cells. Y-axis: normalized read counts. ***indicates *P*<0.0001.

Mixture-of-Isoforms algorithm (MISO), a probabilistic framework that enables quantification of the expression level of alternatively spliced genes and identification of differentially regulated isoforms from RNA-Seq data [18], was used to detect differentially expressed isoforms induced by butyrate in bovine epithelial cells. In this study, MISO detected a total of 216 isoforms from 98 genes that were differentially regulated by butyrate using a combined cutoff of ΔΨ >0.20 (i.e., the posterior distribution over the change in Psi for each event) and Bayes factor >10. The Bayes factor represents the weight of the evidence in the data in favor of differential expression. For example, Bayes factor =2 means that the isoform is two times more likely to be differentially expressed. Of these events, 50 isoforms from 36 genes were supported by at least 3 pair-wise comparisons (Table 4); 29 of the 36 genes had two mRNA isoforms while the rest had at least 3 isoforms. The mRNA-level expression of defensin, beta 1 gene (DEFB1) was not detected in control cells but significantly up-regulated by butyrate (FDR <0.001). This gene had two isoforms. Isoform #1 had 3 exons and was not detectable in control cells but barely detectable in butyrate-treated cells. However, Isoform#2 had 2 exons and was strongly induced by butyrate (Fig. 1). Similarly, IL-18 (ENSBTAG00000000277) had 2 isoforms with the same number of exons. These 2 isoforms differed in the exon structure, nevertheless. Isoform#1 had a similar number of assigned sequence reads while Isoform#2 had approximately 10 times more assigned reads in butyrate-treated cells (252.08±30.51, mean ± sd) than in control cells (25.94±13.69) (*P*<0.0001, Fig. 2). Isoform #1 of origin recognition complex, subunit 1 (ORC1, transcript# ENSBTAT00000052505) had mean read counts of 168.26±52.75 (± sd) and 1239.75±48.02 in butyrate and control groups, respectively, and was strongly repressed by butyrate. Isoform#2 of ORC1 remained unchanged. Histone deacetylase 10 (HDAC10) had 2 isoforms with the read count ratio between dominant (ENSBTAT00000014602) and minor (ENSBTAT00000042583) isoforms greater than 150 in the control cells. Similarly, the dominant isoform was significantly repressed (*P*<0.00001), while the minor isoform was not affected by butyrate. However, the expression of both isoforms of HDAC5 was strongly enhanced by butyrate treatment (data not shown). For genes with ≥3 isoforms, one or 2 isoforms were often differentially regulated by butyrate. As Figure 3 shows, the expression level of Isoforms# 1 and 3 of aminoacylase 1gene (ACY1) was unchanged but that of Isoform#2 was strongly repressed by butyrate (Fig. 3). On the other hand, Isoform#2 of coiled-coil domain containing 24 (CCDC24) was little changed in response to butyrate treatment while Isoforms#1 and 3 were significantly induced by butyrate (Fig. 4). Moreover, differential expression of 6 selected transcript isoforms induced by butyrate, including transcripts such as ENSBTAT00000009005 (LGALS9), ENSBTAT00000010687 (CLSTN3), ENSBTAT00000012035 (MX1), ENSBTAT00000020401 (NASP), ENSBTAT00000036545 (GUK1), and ENSBTAT00000061158 (TACC2), were confirmed using RT-PCR.

## Discussion

Butyrate is one of important short-chain fatty acids (SCFAs). Produced in the rumen and hindgut by gut microorganisms, butyrate is rapidly absorbed and utilized by the rumen and colon epithelium, contributing to 75% of the total energy requirement in ruminants and ∼10% for humans [9], [19]. Additionally, butyrate is shown to reinforce intestinal barriers and modulate motility and visceral sensitivity of the intestine [20]. As a signaling molecule, butyrate induces apoptosis and inhibits cell proliferation, and therefore, it has antitumorigenic properties. Butyrate is an inhibitor of histone deacetylases (HDACs), one of three classes of enzymes epigenetically modifying chromatin histones. Mounting evidence suggests histone acetylation plays a major role in controlling transcriptional activities of genes [21], [22]. HDAC inhibitors such as butyrate induce hyperacetylation of histones, and therefore increase transcriptional activities [2]. This was supported by our observation that the total number of genes transcribed in butyrate-treated cells (mean ± sd =19,322±155) was significantly higher than in untreated control cells (17,626±125) (*P*<0.00001). Transcriptional effects of butyrate have been extensively investigated in various cell types, in both *in vitro* and *in vivo* models [19], [23]–[25]. Indeed, previous studies have demonstrated that butyrate down-regulates genes controlling cell proliferation *in vitro*, resulting in the inhibition of the proliferation of epithelial cells [25], [26]. Microarray results show that butyrate has a profound effect in global gene expression, including up-regulation of genes related to apoptosis and differentiation [10], [26]. Our recent transcriptome studies using RNA-seq technology demonstrate that a 24-h butyrate treatment significantly affects the transcript abundance of 11,408 of the 17,625 genes detected in the bovine epithelial cell, representing ∼65% of the entire transcriptome [11], [19]. However, these studies focus on the transcription at a gene level. The effect of butyrate on individual transcript isoforms and alternative splicing has been systematically studied only recently in human cells [27]. In this study, we examined the regulation of alternative splicing by butyrate in bovine epithelial cells. Our results should facilitate a better understanding of alternative splicing in the development of epithelial cells-derived diseases.

Of four classes of histone deacetylases, butyrate inhibits enzymatic activities of most HDACs in Class I, II, and IV, which are zinc-dependent, except HDAC6 and HDAC10 [28]. Class III HDACs (also called sirtuins or SIRTs) depend on nicotinamide adenine dinucleotide for their catalytic activity [29]. SIRTs are associated with chromtain regulation and affect genome stability in yeast and may represent pivotal regulators of lifespan and aging [30]. SIRTs catalyze two major biochemical reactions: deacetylation on lysine residues of target proteins by altering cellular [NAD^+^]/[NADH] ratios (SIRT1, SIRT2, SIRT3, SIRT5, and SIRT7) and ADP-ribosylation (SIRT4 and SIRT6) [31]. In neuronal cells, SIRT1, SIRT5, and SIRT6 are down-regulated, whereas SIRT2, SIRT4, and SIRT7 up-regulated by butyrate [32]. Our RNA-seq data suggest that butyrate regulated the transcript abundance or gene expression of the majority of HDACs (Table 5). Butyrate significantly increased the expression of HDAC3, HDAC5, and HDAC11. On the other hand, the expression level of HDACs7-10 was significantly down-regulated. The mRNA levels of SIRT4 and SIRT6 were strongly up-regulated while SIRT1 was significantly down-regulated by butyrate (Table 5). However, the relative abundance of HDAC1, HDAC2, HDAC4, and HDAC6 remained unchanged by butyrate. In addition to its effect on the expression at the whole gene level, butyrate selectively regulated the transcript abundance of different mRNA isoforms. While the abundance of both short and long isoforms of HDAC5 was significantly enhanced by butyrate, only the long and dominant isoform of HDAC10 was significantly down-regulated. Intriguingly, butyrate is unable to inhibit the catalytic activity of HDAC10 [28]. Nevertheless, butyrate may still exert its control on the deacetylase activity of HDAC10 via transcriptional regulation at the mRNA level. Our future work will focus on the biological relevance of various HDAC isoforms induced by butyrate, especially various SIRTs and their roles in cell senescence and aging.

**Table 5:** The expression of histone deacetylases affected by butyrate in the bovine epithelial cells.

Ensembl Gene ID	Class	Symbol	Fold	*P*_value	FDR
ENSBTAG00000012698	I	HDAC1	0.96	3.51E–01	0.4154
ENSBTAG00000011849	I	HDAC2	1.04	4.07E–01	0.4718
ENSBTAG00000017360	I	HDAC3	1.56	5.98E–13	0.0000
ENSBTAG00000017764	IIA	HDAC4	0.98	8.09E–01	0.8479
ENSBTAG00000016254	IIA	HDAC5	1.91	0.00E+00	0.0000
ENSBTAG00000013244	IIB	HDAC6	1.02	7.45E–01	0.7934
ENSBTAG00000026819	IIA	HDAC7	0.56	0.00E+00	0.0000
ENSBTAG00000046092	I	HDAC8	0.38	0.00E+00	0.0000
ENSBTAG00000003808	IIA	HDAC9	0.22	0.00E+00	0.0000
ENSBTAG00000011000	IIB	HDAC10	0.50	0.00E+00	0.0000
ENSBTAG00000007208	IV	HDAC11	22.63	0.00E+00	0.0000
ENSBTAG00000014023	III	SIRT1	0.54	0.00E+00	0.0000
ENSBTAG00000001776	III	SIRT2	1.13	9.96E–03	0.0152
ENSBTAG00000002044	III	SIRT3	1.38	5.48E–04	0.0010
ENSBTAG00000021168	III	SIRT4	2.03	8.18E–12	0.0000
ENSBTAG00000014904	III	SIRT5	1.28	1.32E–06	0.0000
ENSBTAG00000019909	III	SIRT6	1.96	0.00E+00	0.0000
ENSBTAG00000000039	III	SIRT7	1.16	1.68E–02	0.0248

Fold changes are expressed as mean RPKM ratios of butyrate treated cells to untreated control cells calculated using the Cuffdiff algorithm. The value in Fold greater than 1.0 indicates a up-regulation by butyrate while the value smaller than 1.0 suggests a down-regulation of the gene expression by butyrate.

Distant gene fusion events are well known in tumors, often resulting from genomic abnormalities such as chromosomal translocation. These events, such as BCL-ABL, lead to the formation of a novel chimeric protein with different functions and are one of the common mechanisms for oncogene activation [33]. Recently, a new type of fusion involving two adjacent genes in the same orientation on the same chromosome has been described [34], [35]. Adjacent genes are normally transcripted independently. However, a single transcript can be occasionally formed to include at least part of one exon from each of two or more distinct genes [36]. This phenomenon, Transcription Induced Chimeras (TICs) [35] or Conjoined Genes (CGs) [36], is widespread in mammalian genomes. It is estimated that at least 4%-5% of the tandem gene pairs in the human genome can be transcripted into TICs. Moreover, these TICs may possess novel functions because >70% of them are conserved in other vertebrate genomes [36]. In this study, we detected 13 TICs that were supported by multiple junction reads. Intriguingly, these TICs were also differentially regulated by butyrate in bovine epithelial cells. Approximately 46% of these fusion events were involved in the members of gene families, which is much higher than 11% as previously reported [36]. For example, TICs were formed between 2 homeobox genes, HOXA6 and HOXA5 and between 2 keratin genes, KRT8 and KRT4. In addition, the fusion between zinc finger proteins ZNF865 and ZNF524 was also supported by multiple junction reads, and a significantly higher number of reads was detected in untreated control cells than in butyrate-treated cells. A similar fusion event between ZNF649 and ZNF577 was identified in prostate tumors [37]. A relatively higher percentage of TICs between genes with related functions identified in this study should be further examined. Most importantly, the functional significance of these fusion events, especially their possible role in transcription regulation, should be experimentally determined.

One advantage of GSNAP algorithm is its potential to identify novel splice sites and therefore possibly novel transcript isoforms. The algorithm relies on a maximum entropy model and uses frequencies of nucleotides neighboring a donor and acceptor splice site to discriminate between true and false splicing sites [14]. The power of this approach was exemplified by a case study involving the prohibitin gene (PHB). The prohibitin protein complex, located in mitochondrial inner membrane, is formed by heteromeric binding of both PHB and PHB2 [38] and is involved in transcription regulation and cell cycle progression by blocking the G1/S transition of the cell cycle [39]. Prohibitin induces apoptosis by interacting with the retinoblastoma protein as well as being involved in the *p53* pathway. Its 3′ untranslated region (UTR) acts as a novel class of non-coding regulatory RNAs. Additionally, PHB expression is up-regulated in the retina in aging and diabetic models and may serve as an oxidative marker [38]. A recent study using thyroid tumor cell lines demonstrates that butyrate increases PHB mRNA expression. Furthermore, butyrate as well as other HDAC inhibitors, such as trichostatin A, affects PHB splicing [2], leading to the over-expression of the longer isoform with 3′ UTR. Both inhibitors decrease the mRNA levels of the shorter isoform but increase those of the longer isoform, which exerts a growth-suppressive action. Our results showed that butyrate significantly down-regulated the mRNA expression of both PHB and PHB2 in the bovine epithelial cell (FDR <0.0001). No known isoforms in both genes have been annotated in cattle so far. Annotated PHB and PHB2 genes have 7 and 10 exons, respectively. GSNAP correctly identified all normally splicing exon-exon junctions. Moreover, GSNAP detected novel splice sites. For example, several junction reads detected in the untreated control cells suggest a possible exon skipping event that may result in skipping of Exon#2 in PHB2. Such reads were not detectable in the butyrate-treated cells. In the PHB gene, significantly higher numbers of junction reads in the control group than the butyrate-treated group indicated that multiple alternative splicing events involved Exon#1 and Exon#2 and the intron between them. These events occurred in the 5′ UTR and did not seem to alter its primary protein structure. In humans, the 3′ UTR of PHB is attributed to its anti-tumorigenic and anti-proliferative properties [40]. The biological implication of various splicing events in the 5′ UTR of PHB genes in cattle is worthy of further investigation.

## Materials and Methods

### 1. Treatments

The bovine epithelial cells used in this study were previously described [11]. The culture and sodium butyrate treatment was essentially the same as reported [26]. Briefly, cells were treated with 10 mM sodium butyrate for 24h. As a result of butyrate treatment, cell cycle arrest was notable. The percentage of G1/G0 cells was increased from ∼41% in normal cell populations to 79% in butyrate-treated cell populations, in a good agreement with previous results [10]. Cells were then harvested and high-quality total RNA (RNA Integrity number or RIN >9.0) was processed using an Illumina TruSeq RNA sample prep kit following the manufacturer’s instruction (Illumina, San Diego, CA, USA). After quality control procedures, individual RNA-seq libraries were then pooled based on their respective sample-specific 6-bp adaptors and sequenced at 50bp/sequence read using an Illumina HiSeq 2000 sequencer as described previously [41]. The mean number of sequence reads generated per sample was 67,527,111±8,330,388.58 (± sd). A total of eight replicates in two groups, butyrate treated (BT) and untreated (control or CT; *N* =4 for each group), were used. Raw sequence reads were deposited to the NCBI Sequence Read Archive (accession# SRA051007.1).

### 2. Data Analysis and Bioinformatics

Raw sequence reads were first checked using our quality control pipeline. Nucleotides of each raw read were scanned for low quality and trimmed using SolexaQA [42]. Input reads were then aligned to the bovine reference genome using GSNAP [14] with default parameters. Two mismatches were tolerated (“-m 2″ flag). The intron size was specified by “-w” =20,000 bp. Read counts, including junction reads, were extracted from GSNAP –N flag output files and further analyzed.

For differentially expressed isoform detection, Mixture-of-Isoforms program (MISO v 0.4.1, released February 1, 2012) [18] was used. Trimmed reads were first aligned to the bovine genome (UMD3.1) with TopHat [43] using default parameters. The TopHat SAM output files were converted into BAM files and input into MISO with the GTF file from ENSEMBL bovine genebuild release 65. Differentially expressed isoforms were detected using the following filtering parameters: the sum of inclusion and exclusion reads is greater than 10 (≥1 inclusion read and ≥1 exclusion read), ΔΨ greater than 0.20, and the Bayes factor ≥10. In addition, each SAM output file from TopHat alignment, along with the GTF file from ENSEMBL bovine genebuild release v65, were used in the Cuffdiff program [44] in the Cufflink package (v1.3.0) to test for differential expression at the gene level, as described previously [19].

### 3. Quantitative RT-PCR

Ten selected alternative splicing events, including 2 exon-skipping event, 2 gene fusion events, and 6 transcript isoforms, were tested using real-time RT-PCR as described previously [45]. Briefly, the cDNA synthesis was performed using an iScript cDNA Synthesis kit (Bio-Rad, Hercules, CA). Real-time RT-PCR analysis was carried out with an iQ SYBR Green Supermix kit (Biorad) using 200 nM of each primer and the 1^st^-strand cDNA (100 ng of the input total RNA equivalents) in a 25 µl reaction volume. The amplification was carried out on an iCycler iQ™ Real Time PCR Detection System (BioRad) with the following profile: 95°C –60s; 40 cycles of 94°C–15s, 60°C –30s, and 72°C –30s. A melting curve analysis was performed for each primer pair. PCR products were further analyzed on a high-sensitive DNA chip using an Agilent Bioanalyzer 2100 (Agilent, Palo Alto, CA, USA) for product length.
